# Functional brain network analysis in patients with upper-limb spasticity after stroke

**DOI:** 10.3389/fnhum.2025.1713235

**Published:** 2025-12-15

**Authors:** Fangwen Gao, Man He, Xubo Hou, Lijie Gou, Kuihua Li, Jinyu Zheng

**Affiliations:** 1 Department of Biomedical Engineering, Chengde Medical University, Chengde, Hebei, China; 2 Department of Rehabilitation Medicine, Affiliated Hospital of Chengde Medical University, Chengde, Hebei, China; 3 Department of Neurology, Affiliated Hospital of Chengde Medical University, Chengde, Hebei, China

**Keywords:** brain networks, electroencephalogram, functional connectivity, graph theroy, stroke, upper-limb spasticity

## Abstract

**Introduction:**

Stroke ranks as the second leading cause of death and disability worldwide, with the resulting upper-limb spasticity severely impairing patients’ motor function and quality of life. However, existing clinical assessment scales exhibit a degree of subjectivity, and research into the neurophysiological mechanisms underlying spasticity remains insufficient. Brain network analysis offers a novel perspective for investigating the neural mechanisms associated with spasticity.

**Methods:**

Eight patients with upper limb spasticity due to stroke (MAS grades 1–2) and eight healthy controls were enrolled. Multi-channel EEG signals were recorded during different upper limb movements (fist clenching, elbow flexion, wrist flexion). Functional brain networks were constructed using the weighted phase delay index, and further calculations were performed on relevant brain network characteristics, including node degree, global efficiency, local efficiency, clustering coefficient, and small-world properties.

**Results:**

Research findings indicate that functional connectivity in spasticity patients is significantly lower than in healthy subjects, particularly in the alpha and beta frequency bands, with weaker cross-regional synchrony in frontal, central, and temporal lobe regions. Graph theory analysis further reveals that compared to healthy controls, spasticity patients exhibit significantly reduced global efficiency, local efficiency, and clustering coefficient, while small-world properties remain relatively preserved. Node degree analysis revealed abnormal compensatory activation in temporal and parietal regions, whereas healthy participants exhibited higher node degrees in central and frontal areas. These findings suggest that spasticity is associated with impaired local and global network integration, accompanied by inefficient compensatory mechanisms.

**Discussion:**

This study provides new evidence that post-stroke upper limb spasticity is not only a peripheral muscle phenomenon but also reflects disturbances in cortical network dynamics. Brain network metrics, particularly global and local efficiency, may serve as objective biomarkers to quantify spasticity severity and guide personalized rehabilitation interventions, offering a promising direction for developing precision rehabilitation strategies.

## 1 Introduction

Stroke ranks as the second leading cause of death and the second leading cause of disability worldwide, with its resulting long-term functional impairments significantly diminishing patients’ quality of life (Feigin et al., 2021). Upper limb muscle spasticity, one of the most common complications following stroke (Ward, 2008), clinically manifests as abnormally elevated muscle tone on the hemiplegic side, altered muscle group coordination patterns, and loss of voluntary motor control. This leads to motor dysfunction such as weakened grip strength and restricted joint mobility (Milani et al., 2022). Common clinical assessment methods include the Ashworth Scale (Wissel et al., 2013), Fugl-Meyer Assessment (Lance, 1980), and Modified Ashworth Scale (MAS) (Pandyan et al., 1999). However, these traditional scales have limitations. First, their evaluation relies heavily on the subjective judgment of rehabilitation therapists or physicians, with limited inter-rater reliability, leading to assessment discrepancies among different clinicians. Second, these scales typically employ a 5- to 6-level grading system with low resolution, making it difficult to capture subtle variations in spasticity severity. More critically, traditional clinical scales fail to reveal the neurophysiological mechanisms underlying spasticity, thereby hindering the identification of precise neurological targets for targeted rehabilitation interventions. This situation underscores the urgent need within rehabilitation medicine for objective, quantitative assessment methods that reflect central nervous system mechanisms. Consequently, objective detection and evaluation techniques such as electrophysiology and functional magnetic resonance imaging (fMRI) are increasingly being applied to the study of post-stroke spasticity.

In recent years, functional connectivity analysis has been widely applied in research on various neurological disorders. Among these methods, the weighted Phase Lag Index (wPLI) improves upon traditional coherence metrics by reducing bias caused by volume conduction. Compared to conventional coherence measures, wPLI effectively mitigates the impact of volume conduction effects while placing greater emphasis on phase difference consistency. This approach better reflects genuine neural activity and minimizes spurious connectivity. This makes wPLI a more reliable measurement method for assessing interactions between EEG channels. Yan et al. (2021) further validated wPLI’s advantage in revealing brain network abnormalities by analyzing functional connectivity differences between Alzheimer’s disease patients and healthy controls across different frequency bands using wPLI parameters.

With advances in neuroelectrophysiology, researchers have gradually shifted their focus from lesioning isolated regions to the remodeling of entire brain networks (Bistriceanu et al., 2023). The introduction of complex network theory has provided new insights into studying post-stroke brain dysfunction (Asadi et al., 2023). By abstracting brain regions as nodes and functional connections between regions as edges, functional brain networks can be constructed. Graph theory metrics are then used to quantify their topological characteristics. These metrics—including global efficiency, local efficiency, clustering coefficient, small-world properties, and node degree—reflect the brain’s integration and separation capabilities during information processing. Lin et al. (2023) investigated changes in brain connectivity and network topology by recording EEG during active, passive, and myocardial infarction conditions while patients in the subacute and chronic phases of stroke performed handgrip movements. They also measured outcomes using the Fugl-Meyer Upper Limb Assessment Scale and the Modified Ashworth Scale. In epilepsy research, Abbas et al. proposed a functional connectivity network estimation method based on EEG and applied it to neonatal seizure analysis, offering new insights into abnormal brain network activity (Abbas et al., 2021). Alaei et al. (2023) employed EEG source reconstruction and graph theory methods for directed brain network analysis, revealing significant differences in network topological features between anxiety-type and non-anxiety-type depression. These studies further demonstrate that brain network analysis offers a powerful tool for understanding the mechanisms underlying different diseases.

Electroencephalogram (EEG) has been widely applied in brain function research due to its high temporal resolution and strong operability (Zhang et al., 2023). For instance, Van Kaam et al. (2018) demonstrated significant alterations in ipsilateral and contralateral brain activity and functional connectivity during resting-state EEG in acute stroke patients. Compared to functional magnetic resonance imaging (fMRI), EEG exhibits greater sensitivity in capturing transient dynamic changes associated with motor tasks. This capability was validated in studies examining beta-wave cortical-muscle phase synchronization in stroke patients with hemiplegia.

Therefore, this study investigated the brain network characteristics of post-stroke patients with upper-limb spasticity during three motor tasks: fist clenching, elbow flexion, and wrist flexion. For each task, functional connectivity matrices were constructed based on the weighted Phase Lag Index (wPLI) computed across delta, theta, alpha, and beta frequency bands. Graph-theoretical measures were then derived from these matrices to quantify brain network organization, and comparisons were conducted between the patient and healthy control groups. The study aimed to elucidate task-specific and frequency-dependent alterations in brain network topology associated with spasticity, providing objective neurophysiological markers for spasticity severity. These analyses are expected to advance the understanding of the neural mechanisms underlying the occurrence and maintenance of spasticity and to inform potential individualized rehabilitation strategies based on brain network features.

## 2 Materials and methods

### 2.1 Participants

As an exploratory pilot study, this research included 16 participants, comprising 8 patients (aged 39–68 years) with upper limb muscle spasticity following stroke and 8 healthy controls (aged 21–48 years). All patients were recruited from the Department of Rehabilitation Medicine at Chengde Medical University Affiliated Hospital. The study protocol was approved by the Chengde Medical University Ethics Committee (No.: 2024027) and strictly adhered to the ethical requirements of the Declaration of Helsinki. All participants voluntarily enrolled after providing informed consent. Inclusion criteria for the patient group were: first-time unilateral ischemic stroke with stable condition; spasticity in the affected upper limb with Modified Ashworth Scale (MAS) grade of 1, 1+, or 2; age between 20 and 65 years; and ability to comprehend and cooperate with experimental tasks. Exclusion criteria included comorbidities affecting muscle tone such as Parkinson’s disease, spinal cord injury, myasthenia gravis, or polymyositis; prior antispasticity treatments (e.g., oral antispasmodics, botulinum toxin injections, local blocks, or surgery); MAS grade 4 (muscle rigidity preventing movement); neurological disorders affecting EEG signals (e.g., epilepsy, encephalitis, brain tumors, migraine), and severe cognitive or psychiatric disorders. Healthy controls were right-handed with no history of neurological, psychiatric, or orthopedic conditions. Among the 8 enrolled patients, 7 had left-sided spasticity and 1 had right-sided spasticity (Table 1). To ensure consistency in data analysis, we employed a mirroring technique to process EEG data from right-sided spasticity patients, uniformly treating them as “left-sided hemiplegia.” This method has been validated and applied in previous studies (Su et al., 2025).

**Table 1:** Patient information.

Subject No.	MAS		Affected (Side)	Age (years)	Gender M/W	Time since stroke onset (months)
	Upper limbs	Hand				
Sub 01	0	1+	L	54	M	1
Sub 02	1+	1	L	39	M	1
Sub 03	0	1+	L	45	M	3
Sub 04	1+	0	L	68	M	3
Sub 05	1	0	R	61	M	1
Sub 06	1	0	L	39	M	1
Sub 07	1+	1	L	51	W	1
Sub 08	1+	1	L	45	M	3

### 2.2 Experimental paradigm

**Figure 1:**
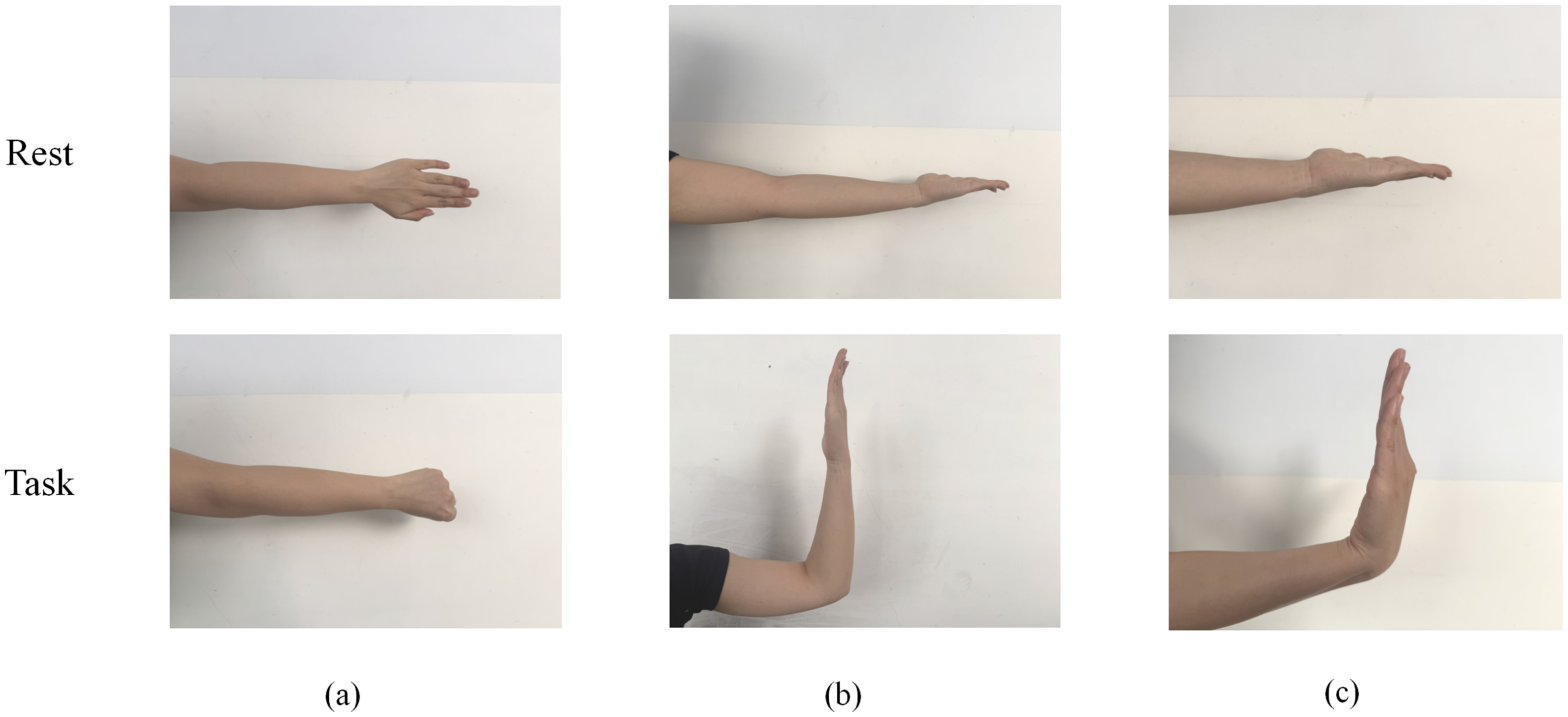
Experimental movement demonstration diagram. **(a)** Fist clenching. **(b)** Elbow flexion. **(c)** Wrist flexion.

**Figure 2:**
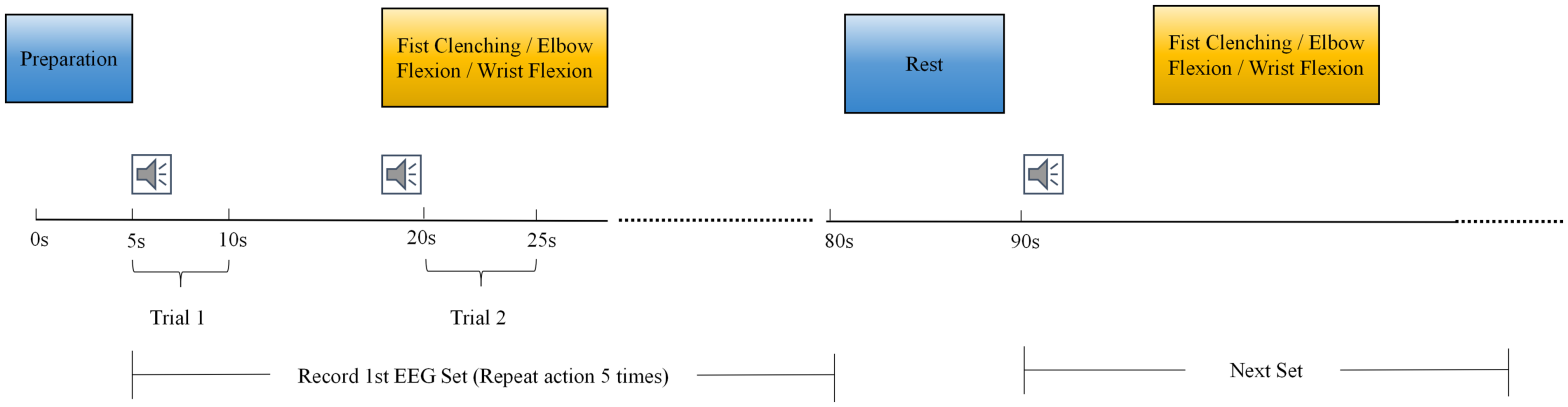
Experimental flowchart. The speaker icon represents the voice command broadcast.

**Figure 3:**
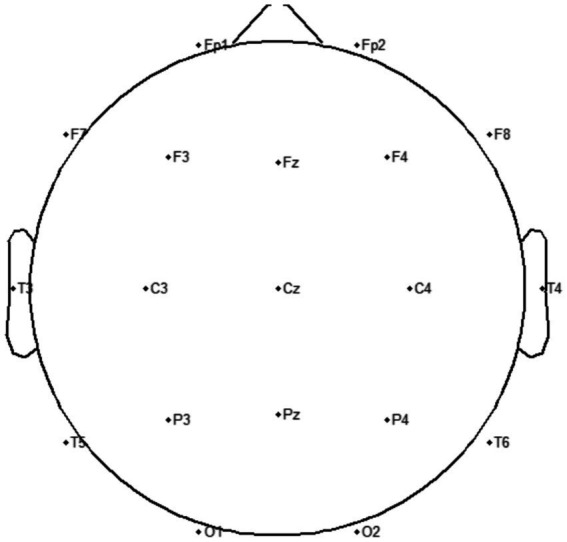
Electrode site map. Frontal region: Core area for higher cognitive control, involving executive function, attention regulation, emotional regulation, motor planning, and language production. Corresponding electrodes: Fp1, Fp2, Fz, F3, F4, F7, F8. Left Temporal Region: Associated with auditory processing, language comprehension, and emotional memory. Corresponding electrodes: F7, T3, T5. Central Region: Covers the sensorimotor cortex, involved in motor and tactile processing. Corresponding electrodes: C3, Cz, C4; Right temporal region: Associated with emotion and auditory processing. Corresponding electrodes: F8, T4, T6; Occipital lobe: Processes visual information. Corresponding electrodes: O1, Oz, O2; Parietal lobe: Primarily responsible for somatosensory integration, spatial perception, attention control, and multimodal information processing. Corresponding electrodes: P3, Pz, P4.

The experiment was conducted in a quiet, soundproof, and shielded laboratory. Air conditioning and lighting were turned off during the experiment to minimize power frequency interference. Participants sat upright in comfortable chairs with eyes closed and upper limbs resting naturally. Two hours prior to the experiment, all participants washed their hair with shampoo and allowed it to air dry naturally to ensure good electrical contact for the EEG electrodes. Prior to data acquisition, EEG gel was applied to maintain impedance below 5 kΩ. The experimental tasks comprised three movement categories: fist clenching, elbow flexion, and wrist flexion (Figure 1). Each movement was initiated immediately upon hearing a verbal cue and sustained for approximately 5 s, with 10-s intervals between movements to prevent fatigue (Figure 2). Each movement was repeated 20 times (four sets of 5 repetitions each). The experiment employed the Neuracle system (Neurofax EEG-1200C, Nihon Kohden Corporation) to collect 21-channel EEG signals, placed according to the international 10–20 system (Figure 3). EEG signals were recorded at a sampling frequency of 1,000 Hz.

### 2.3 Data pre-processing

Electroencephalogram recordings captured brain activity during subjects’ motor intentions (e.g., clenching fists, flexing elbows) and execution of these movements. The effective recording window spanned 5 s (1 s before and 4 s after the action). EEG preprocessing was performed using the EEGLAB toolbox (version 2022.1). Baseline correction was applied using the 1 s pre-movement interval. To remove slow drifts and high-frequency noise while preserving the physiological bands of interest, FIR digital bandpass filters (0.1–45 Hz) and notch filters (49–51 Hz) were applied. The sampling rate was downsampled to 500 Hz to reduce computational load while maintaining a Nyquist frequency well above the highest analyzed band. Data were re-referenced to the linked A1 and A2 electrodes. Artifacts were removed using Independent Component Analysis (ICA) via the runica algorithm. Components reflecting eye movements, cardiac signals, and muscle activity were identified using ICLabel classification (Pion-Tonachini et al., 2019) combined with visual inspection of scalp maps, time courses, and power spectra. To specifically mitigate EMG contamination in the beta band, components exhibiting muscle-like scalp distributions or broad high-frequency spectral characteristics were removed. Finally, the cleaned continuous EEG was segmented, and epochs containing residual muscle bursts or non-stereotypical artifacts were rejected based on visual inspection. The EEG was divided into delta (2–4 Hz), theta (4–8 Hz), alpha (8–13 Hz), and beta (13–30 Hz) frequency bands for analysis.

### 2.4 Brain network construction and metric analysis

Functional brain networks are commonly constructed using various methods such as coherence, Phase Locking Value (PLV), and partial coherence. These approaches can characterize inter-regional interactions from different perspectives and are relatively straightforward to compute; however, they are often susceptible to volume conduction effects and common source interference, which may lead to spurious functional connections. To address these limitations, the present study employed the weighted Phase Lag Index (wPLI) to estimate phase synchronization between EEG signals. The computation procedure included: (i) constructing functional connectivity matrices based on wPLI values between electrode pairs, (ii) applying thresholding to obtain brain functional networks, and (iii) analyzing the topological architecture of the resulting networks using graph-theoretical approaches. The derived network metrics comprised node degree (D), global efficiency (GE), local efficiency (LE), clustering coefficient (CC), and small-world property (SW). This framework enables a systematic quantification of both the integrative and segregated properties of brain functional networks.

#### 2.4.1 weighted Phase Lag Index (wPLI)

The weighted Phase Lag Index (wPLI) quantifies phase synchrony between two neural signals by weighting the imaginary component of the cross-spectrum. This emphasizes stable phase lead or lag relationships between signals, effectively reducing false synchrony estimates caused by common sources or noise (Imperatori et al., 2019). Time–frequency representations were obtained using a short-time Fourier transform (STFT) applied to single-trial EEG data in the 1–30 Hz range (1-Hz steps). We used 0.4 s Hann windows to maximize temporal resolution. For each channel, this produced complex spectra C(f,t) . From these, the cross-spectrum $\;S_{xy}$ between two channels x and y was computed as $S_{xy}=C_{x}C_{y}^{*}$ , where ∗ denotes the complex conjugate.

The wPLI was then calculated using the imaginary part of the cross-spectrum across trials, defined as:


$$wPLI_{xy}=\frac{\left|E\left[\zeta \left(S_{xy}\right)\right]\right|}{E\left[\left|\zeta \left(S_{xy}\right)\right|\right]}$$

Where $\zeta \left(S_{xy}\right)$ denotes the imaginary part of the cross-spectrum, and E[·] denotes the expected value (average) across trials (epochs). The wPLI value ranges from 0 to 1. A higher wPLI value indicates a higher level of phase synchronization between two neural oscillatory signals, while a lower value indicates a lower level (Shafiei et al., 2024). In this study, we employed wPLI as a metric for phase synchronization between nodes in the brain network. Average functional connectivity strength was calculated across the delta, theta, alpha, and beta frequency bands to quantify the overall level of phase synchronization among brain regions within each band. A 19 × 19 functional connectivity matrix was constructed, with all diagonal elements set to zero and symmetry maintained on both sides of the diagonal.

#### 2.4.2 Construction of brain functional networks and threshold selection

For each subject, we computed a 19 × 19 correlation matrix by defining 19 electrodes (“Fp1,” “F3,” “C3,” “P3,” “O1,” “Fp2,” “F4,” “C4,” “P4,” “O2,” “F7,” “T3,” “T5,” “F8,” “T4,” “T6,” “Fz,” “Cz,” and “Pz”) as network nodes and defining the wPLI values between electrodes as edges to construct a weighted undirected network (Ji et al., 2017). For each subject, binary undirected networks were constructed by applying a sparsity threshold to the connectivity matrix. A threshold range of 17 to 47% (in 5% increments) was selected for this study. This specific range was chosen to: (i) ensure that the resulting networks remained fully connected (minimizing graph fragmentation), (ii) avoid the inclusion of excessive spurious connections associated with high densities, and (iii) maintain comparability with prior EEG network studies.

#### 2.4.3 Analysis of brain functional network metrics

The complexity of brain functional network connectivity can be studied using graph theory (Rubinov and Sporns, 2011), a mathematical approach for analyzing networks that simplifies the brain’s intricate networks by representing nodes and edges schematically. The Graph Theory Network Analysis GRETNA toolbox (version 2.0.0) was employed to analyze wPLI-based brain networks (Wang et al., 2015), examining metrics such as node degree, global efficiency, local efficiency, clustering coefficient, and small-world properties.

1. Node Degree (*D*)

The node degree of a node refers to the number of edges connecting it to its adjacent nodes. Its calculation formula can be expressed as:


$$D=\sum_{j\in N}a_{ij}$$

Here, N represents the total number of nodes in the network, and a_ij denotes the element located at row i and column j of the matrix. The higher the node degree, the more connections a node possesses, and the more significant its position within the network becomes.

1. Clustering Coefficient (*CC*)

The clustering coefficient is commonly used to describe the connection density among nodes in a network (Wang et al., 2024). The clustering coefficient of a node represents the likelihood of that node forming connections with other nodes, defined as:


$$CC_{i}=\frac{2E_{i}}{k_{i}\left(k_{i}-1\right)}$$

Here, *E_i_* denotes the number of connections between node *i* and its neighboring nodes, while *k_i_* represents the number of neighboring nodes. The network’s clustering coefficient equals the average clustering coefficient of each node:


$$CC=\frac{1}{N}\Sigma_{i=1}^{N}CC_{i}$$

1. Local Efficiency (*LE*)

*LE* measures how efficiently a network transmits information through connections between adjacent nodes, reflecting the network’s ability to locally transmit information (Li et al., 2024). For a node *i*, *LE_i_* can be calculated as the average efficiency of all nodes within the subgraph formed by *i* and its adjacent nodes. The *LE* of a network is computed as the cumulative average of all *GE_i_* values across all nodes, following a process similar to *GE* calculation. The specific formula is as follows:


$$LE=\frac{1}{N}\sum_{i=1}^{N}LE_{i}=\frac{1}{N}\sum_{i=1}^{N}\frac{\sum_{j,h\in N,j\neq i}a_{ij}a_{ih}{\left[d_{jh}\left(N_{i}\right)\right]}^{-1}}{k_{i}\left(k_{i}-1\right)}$$

Among these, $LE_{i}$ denotes the local efficiency of node *i*, while $d_{jh}\left(N_{i}\right)$ represents the shortest path length between node *j* and node *h*. *LE* can to some extent reflect the network’s fault tolerance capability.

1. Global Efficiency (*GE*)

Reflects the overall efficiency of information transmission within a network. Global efficiency is defined as the average of the reciprocals of the shortest path lengths for all nodes (Ma et al., 2018), calculated using the following formula:


$$GE=\frac{1}{N\left(N-1\right)}\sum_{i\neq j}\frac{1}{L_{ij}}$$

Here, *N* denotes the number of nodes in the network, and $L_{ij}$ represents the distance between nodes *i* and *j*.

1. Small-world property (*SW*)

*SW* serve as a core metric for evaluating the organizational characteristics of functional brain networks. *SW* refer to a network structure exhibiting both a high clustering coefficient and a short average path length (Watts and Strogatz, 1998). Its calculation formula is defined as follows:


$$SW=\frac{C/C_{rand}}{L/L_{rand}}$$

Here, *C* and *L* represent the clustering coefficient and characteristic path length of the constructed brain network, respectively, while *C_rand_* and *L_rand_* denote the clustering coefficient and characteristic path length of the random brain network. When *SW* > 1, the constructed network exhibits small-world properties. Networks with *SW* generally demonstrate robust structural integrity and exhibit high transmission rates and computational capabilities (Fan et al., 2023).

### 2.5 Statistical analysis

The analysis was performed using SPSS version 22.0. First, Shapiro–Wilk normality tests were performed on all variables. For variables meeting normality assumptions, independent samples t-tests were used to compare differences between groups. For variables not meeting normality assumptions, Mann–Whitney U tests or Wilcoxon signed-rank tests were employed. To control for the increased risk of Type I errors associated with multiple comparisons across different frequency bands and network metrics, p -values were adjusted using the False Discovery Rate (FDR) method (Benjamini-Hochberg procedure). The significance level was set at an FDR-adjusted p < 0.05.

## 3 Results

### 3.1 Functional connection (FC)

**Figure 4:**
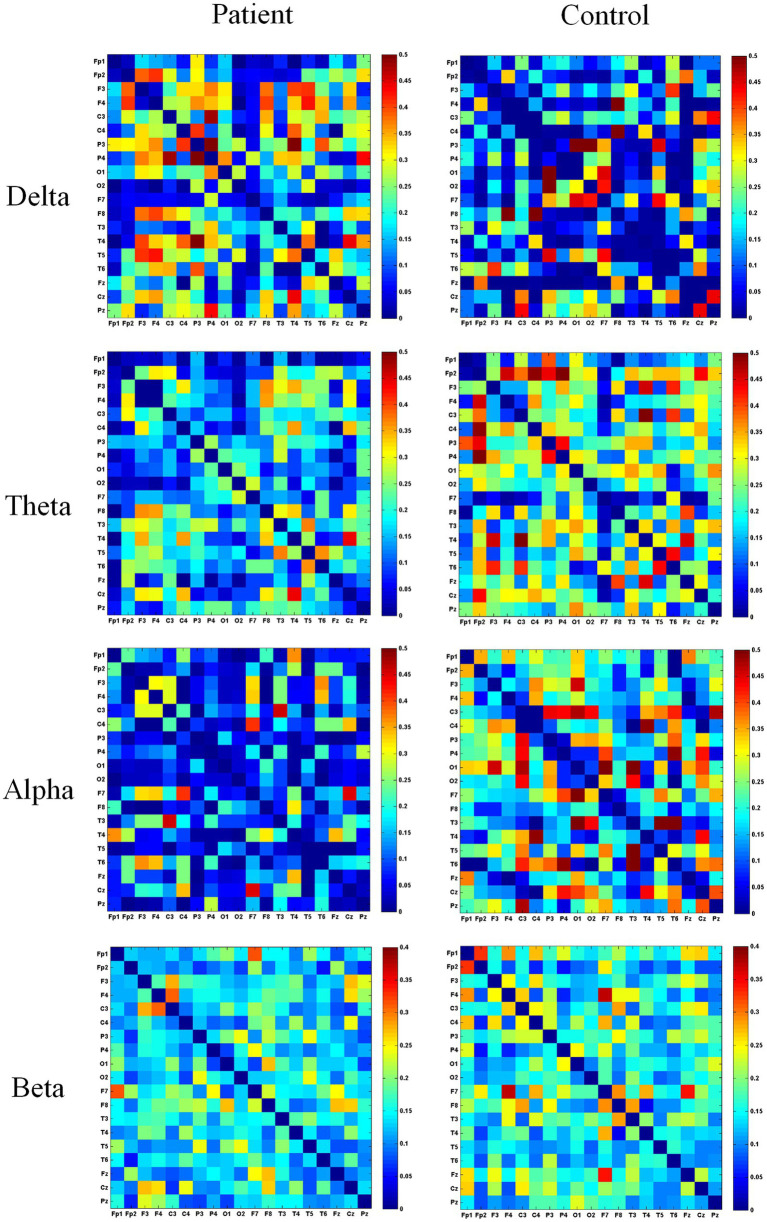
Heatmap of the adjacency matrix in the fist state. This figure depicts the adjacency matrix of *wPLI* values across different frequency bands during fist clenching. “Patient” denotes the patient group, “Control” denotes the healthy control group. Blue indicates a *wPLI* value of 0, while redder hues signify higher *wPLI* values and stronger functional connectivity between electrodes.

This study calculated functional connectivity matrices across four frequency bands (delta, theta, alpha, beta) using EEG data from patient and healthy groups during different movements, with group averages representing their overall levels. The adjacency matrices for both groups during the fist-clenching movement are shown in Figure 4. Adjacency matrices constructed using wPLI were compared between the two groups for specific frequency bands during the fist-clenching state. In the delta band, left hemiplegic patients exhibited stronger functional connectivity at electrodes overlying the occipital (O1), frontal (F7), and temporal (T3) regions during the fist-clenching state, while the healthy group showed stronger connectivity in the frontal, temporal, and occipital regions. In the theta band, left hemiplegic patients demonstrated significantly higher wPLI values at the P3 electrode compared to other sites, whereas the healthy group showed significant connectivity at P3, P4 (parietal lobe), and F3, F4 (frontal lobe). In the alpha band, left hemiplegic patients exhibited higher wPLI values at electrodes associated with the left temporal area (T3) and the central sensorimotor area (C3) compared to right brain regions, indicating stronger functional connectivity. This reflects impaired left-sided limb function and right brain damage, with superior left brain connectivity relative to the right. Healthy subjects exhibited stronger connectivity at T3, T4, T5, T6 (entire temporal region), and C3, C4 during fist clenching. Overall, the healthy group showed stronger functional connectivity at C3, F1, and T3 electrodes compared to the patient group. In the beta band, left hemiplegic patients exhibit higher wPLI values at C3 and F7 electrodes compared to right brain regions. The healthy group demonstrates stronger connectivity at frontal electrodes (F4, F7, Fz) and C3/C4, indicating that the fist-clenching action in the beta band is associated with the premotor cortex and primary motor cortex.

**Figure 5:**
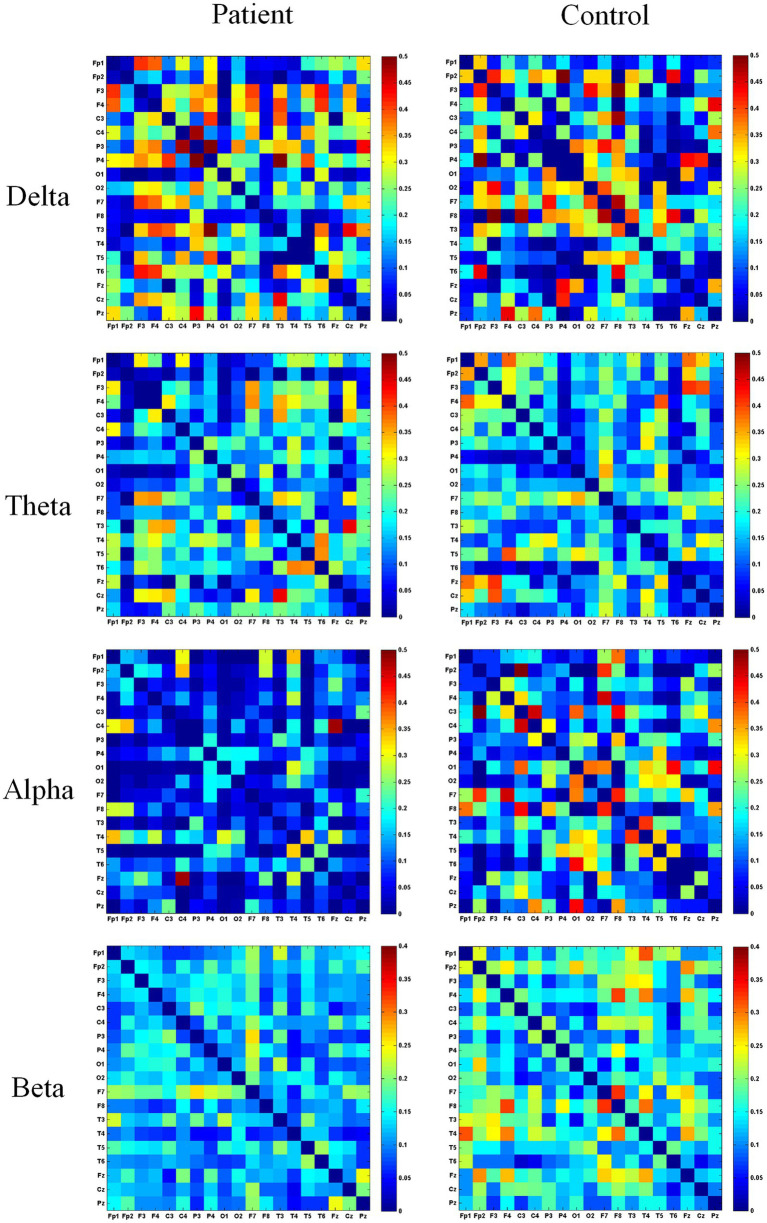
Heatmap of the adjacency matrix in the flexed elbow state. This figure depicts the adjacency matrix of *wPLI* values across different frequency bands during elbow flexion. “Patient” denotes the patient group, while “Control” represents the healthy control group. Blue indicates a *wPLI* value of 0, with redder hues signifying higher *wPLI* values and stronger functional connectivity between electrodes.

The adjacency matrix for the elbow-bent state is shown in Figure 5. In the delta band, the patient group exhibited stronger functional connectivity at the F7 and F8 electrodes, while the healthy group showed stronger connectivity at F7 and F8 (lateral frontal regions). In the theta band, left hemiplegic patients demonstrated stronger functional connectivity at F3 and Fz electrodes, whereas healthy individuals showed stronger connectivity at F3, F4, and Fz (frontal regions). In the alpha band, left hemiplegic patients showed significant connectivity at electrodes C4, F8, and T4, while healthy subjects exhibited higher wPLI values at C3, C4 (central region) and F7, F8 (lateral frontal region). In the beta band, patients with left-sided hemiplegia exhibited stronger functional connectivity at electrodes P3, F7, and T3, while healthy individuals showed stronger functional connectivity at electrodes F7, F8 (lateral frontal), T3, and T4 (temporal region).

**Figure 6:**
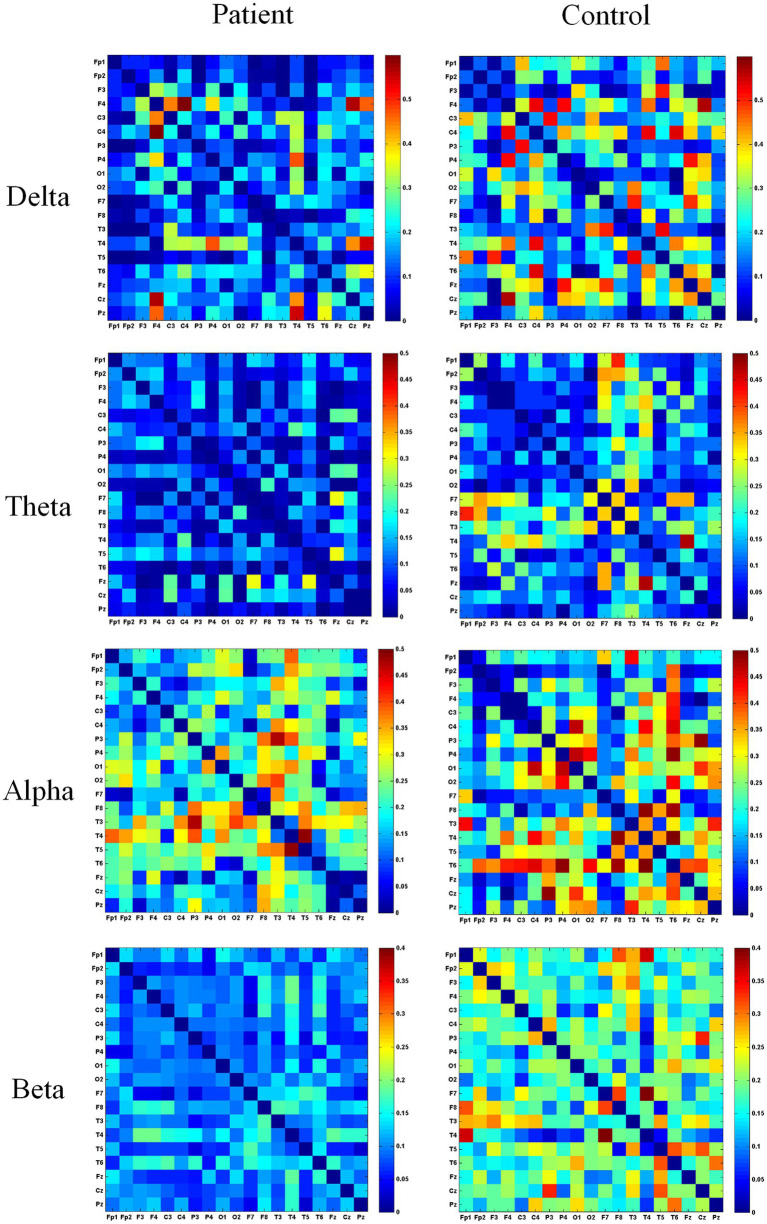
Heatmap of the adjacency matrix in flexed wrist position. This figure depicts the adjacency matrix of *wPLI* values across different frequency bands during wrist flexion. “Patient” denotes the patient group, while “Control” represents the healthy control group. Blue indicates a *wPLI* value of 0, with redder hues signifying higher *wPLI* values and stronger functional connectivity between electrodes.

Figure 6 shows functional connectivity in the wrist flexion state for two groups of subjects. In the delta band, significant connectivity was observed in the wrist flexion state of left hemiplegic patients at the F4, C4, and T4 electrodes. In the delta band, functional connectivity was prominent in the frontal, central, and temporal regions during wrist flexion in healthy subjects. In the theta band, the patient group showed significant connectivity at electrodes F7 and T5. In the alpha band, left hemiplegic patients exhibited pronounced connectivity at T3 and T5, while the healthy group demonstrated significant connectivity at T3, T4, T5, and T6 (temporal region). In the beta band, higher wPLI values were observed at electrodes F8, T4, and T6. The healthy group exhibited stronger functional connectivity at F7, F8 (lateral frontal region), T3, T4, T5, and T6 (temporal region). Overall, connectivity was stronger in the healthy group than in the patient group, particularly in right-brain regions.

### 3.2 Brain network feature analysis

**Figure 7:**
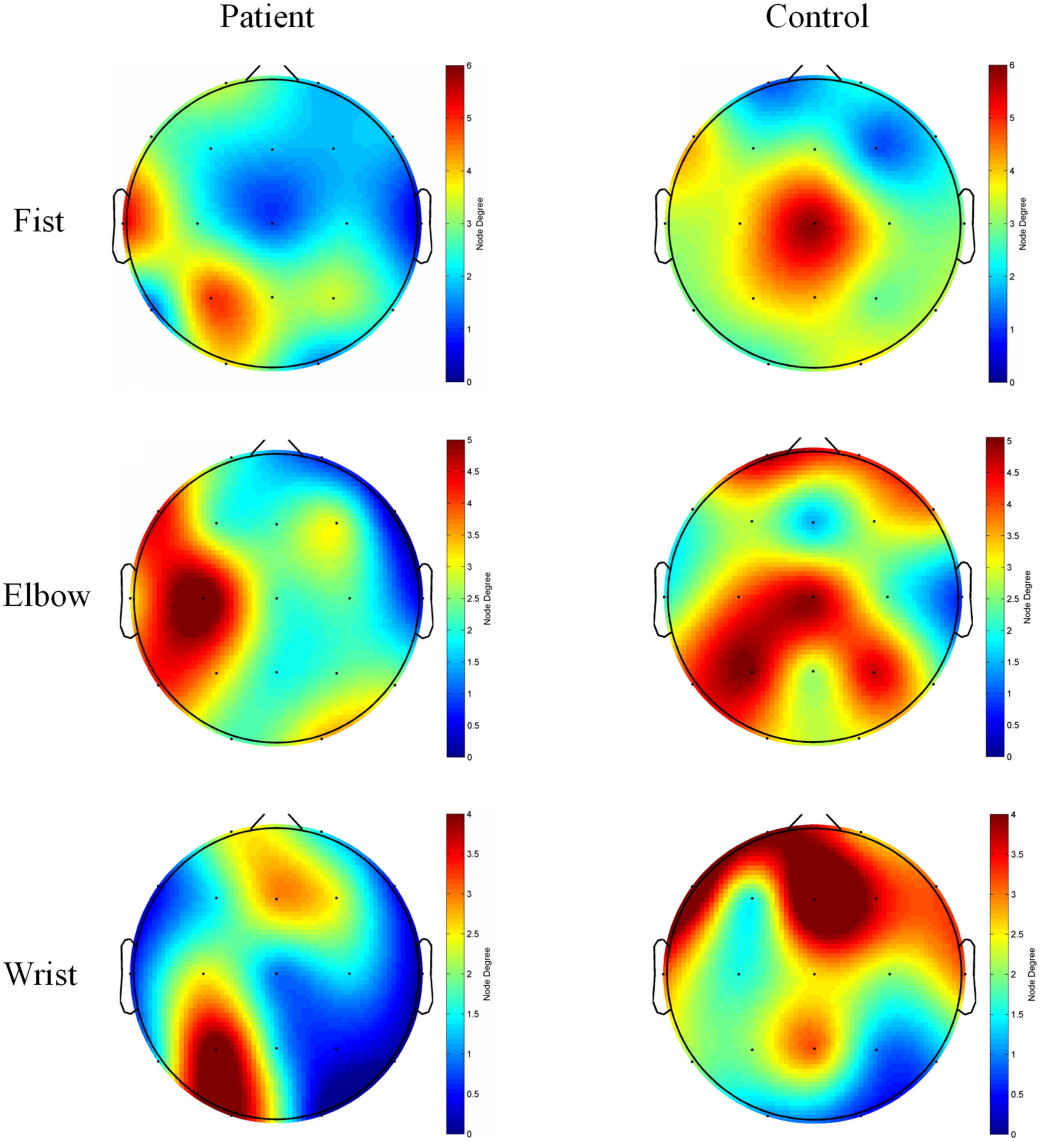
Nodal degree topography of patient group and control group under different actions. This figure shows the node degree topography maps for two groups of subjects performing different actions. “Patient” denotes the patient group, “Control” denotes the healthy group, “Fist” denotes the fist-clenching action, ‘Elbow’ denotes the elbow flexion action, and “Wrist” denotes the wrist flexion action. Higher node degrees are represented by redder colors, while lower node degrees are represented by bluer colors.

Figure 7 shows the node degree maps for the two groups of subjects under different movement states. In the fist-grip state, left hemiplegic patients exhibited higher node degrees at T3 and P3, while healthy subjects showed higher node degrees in the central and parietal regions. During elbow flexion, the patient group exhibited higher nodal degrees at C3 and P3 nodes, while the healthy group showed higher nodal degrees in the central and parietal regions. During wrist flexion, the left-sided hemiplegic patient demonstrated higher nodal degree at P3, while the healthy group exhibited higher nodal degrees in the frontal and parietal regions.

The descriptive statistics for the global network metrics (Global Efficiency, Local Efficiency, Clustering Coefficient, and Small-world property) across the three motor tasks for both groups are summarized in Table 2.

**Table 2:** GE, LE, CC, and SW (mean ± SD) for each task in both groups.

Metric	Task	Patient group (*n* = 8)	Control group (*n* = 8)
Global Efficiency (GE)	Fist	0.29 ± 0.01	0.38 ± 0.04
	Elbow	0.30 ± 0.07	0.38 ± 0.06
	Wrist	0.29 ± 0.05	0.38 ± 0.03
Local Efficiency (LE)	Fist	0.37 ± 0.13	0.41 ± 0.06
	Elbow	0.22 ± 0.05	0.38 ± 0.08
	Wrist	0.31 ± 0.04	0.36 ± 0.06
Clustering Coefficient (CC)	Fist	0.39 ± 0.03	0.50 ± 0.04
	Elbow	0.42 ± 0.07	0.43 ± 0.06
	Wrist	0.43 ± 0.07	0.48 ± 0.04
Small-Worldness (SW)	Fist	0.85 ± 0.08	0.97 ± 0.14
	Elbow	0.90 ± 0.04	0.98 ± 0.09
	Wrist	0.92 ± 0.09	1.00 ± 0.13

**Figure 8:**
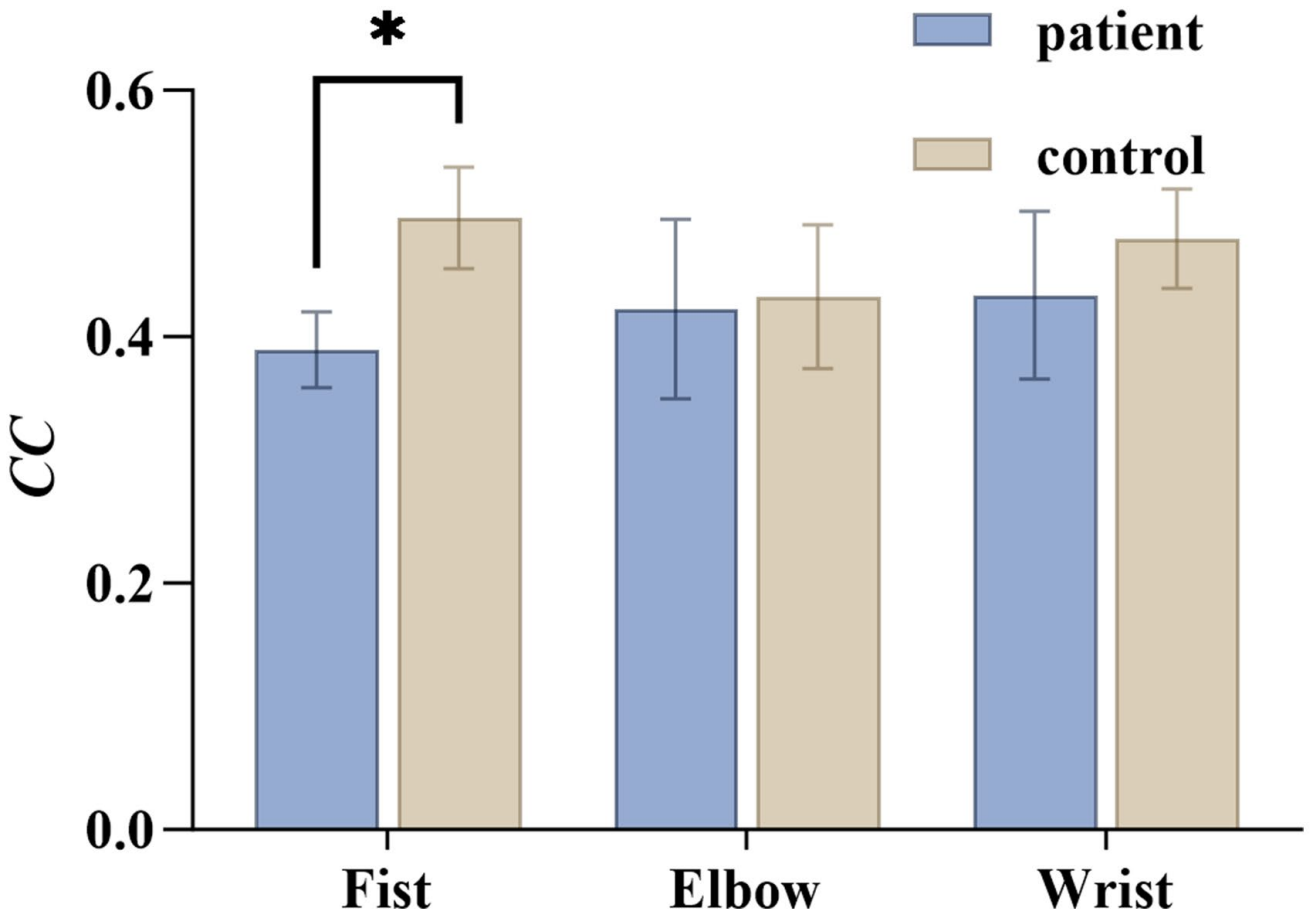
Statistics of clustering coefficients under three actions in the patient and healthy groups. *CC* denotes the clustering coefficient.

Figure 8 shows the differences in clustering coefficients between the two groups under different actions. In the fist-clenching task, the patient group exhibited significantly lower values than the healthy group ( t(14) = 5.187, p<0.001 , $Cohen^{\prime }s\;d=2.59$ ), indicating reduced brain network modularity and impaired local connectivity in the patient group.

**Figure 9:**
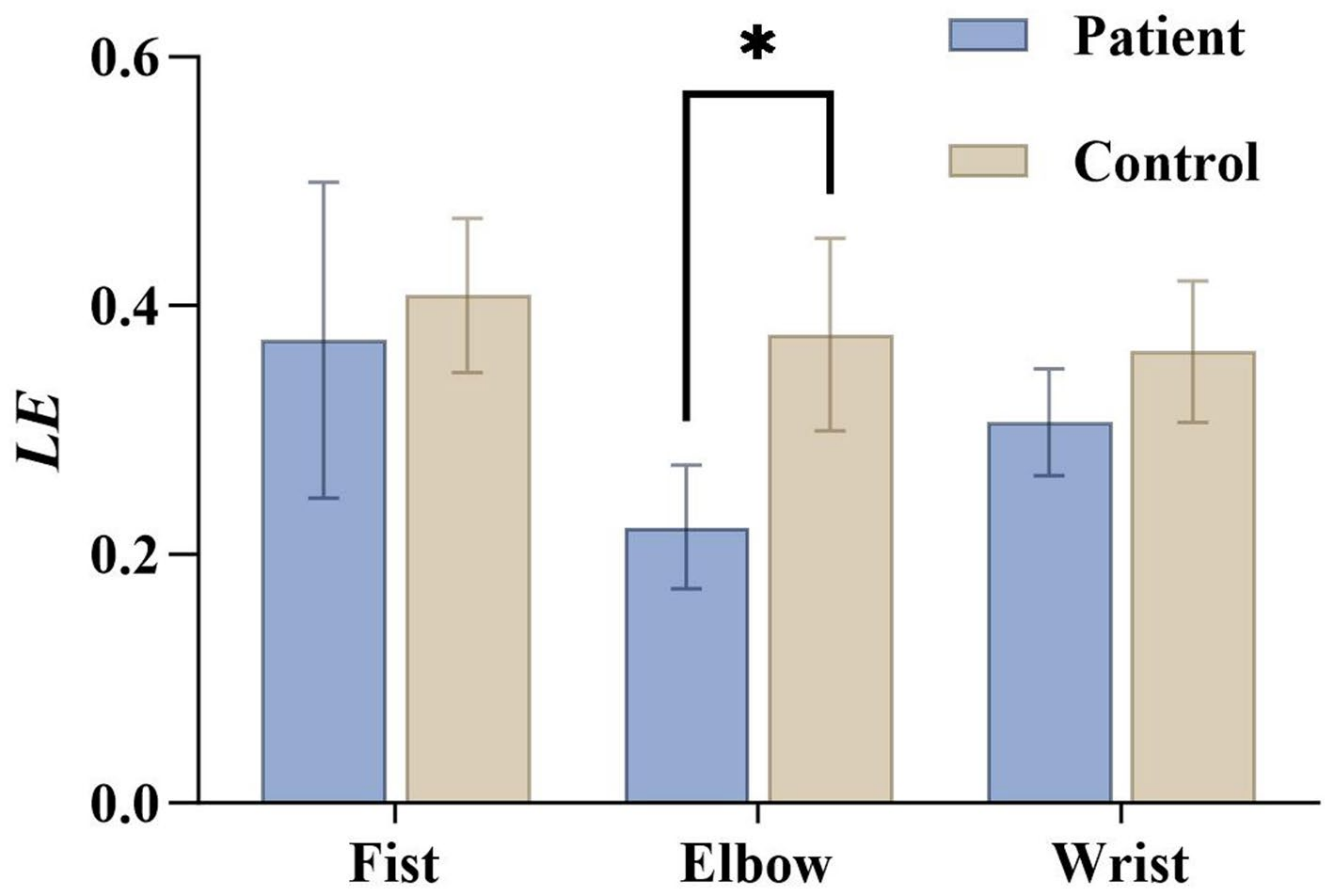
Statistical graph of local efficiency under three maneuvers in the patient group and the healthy group. *LE* denotes local efficiency.

The results of local efficiency (*LE*) (Figure 9) indicate that the patient group exhibited significantly lower values than the healthy group during elbow flexion ( t(14) = 5.321, p<0.05 , $Cohen^{\prime }s\;d=2.66$ ). This suggests impaired local information processing and reduced redundancy mechanisms in patients during this task, leading to decreased network fault tolerance.

**Figure 10:**
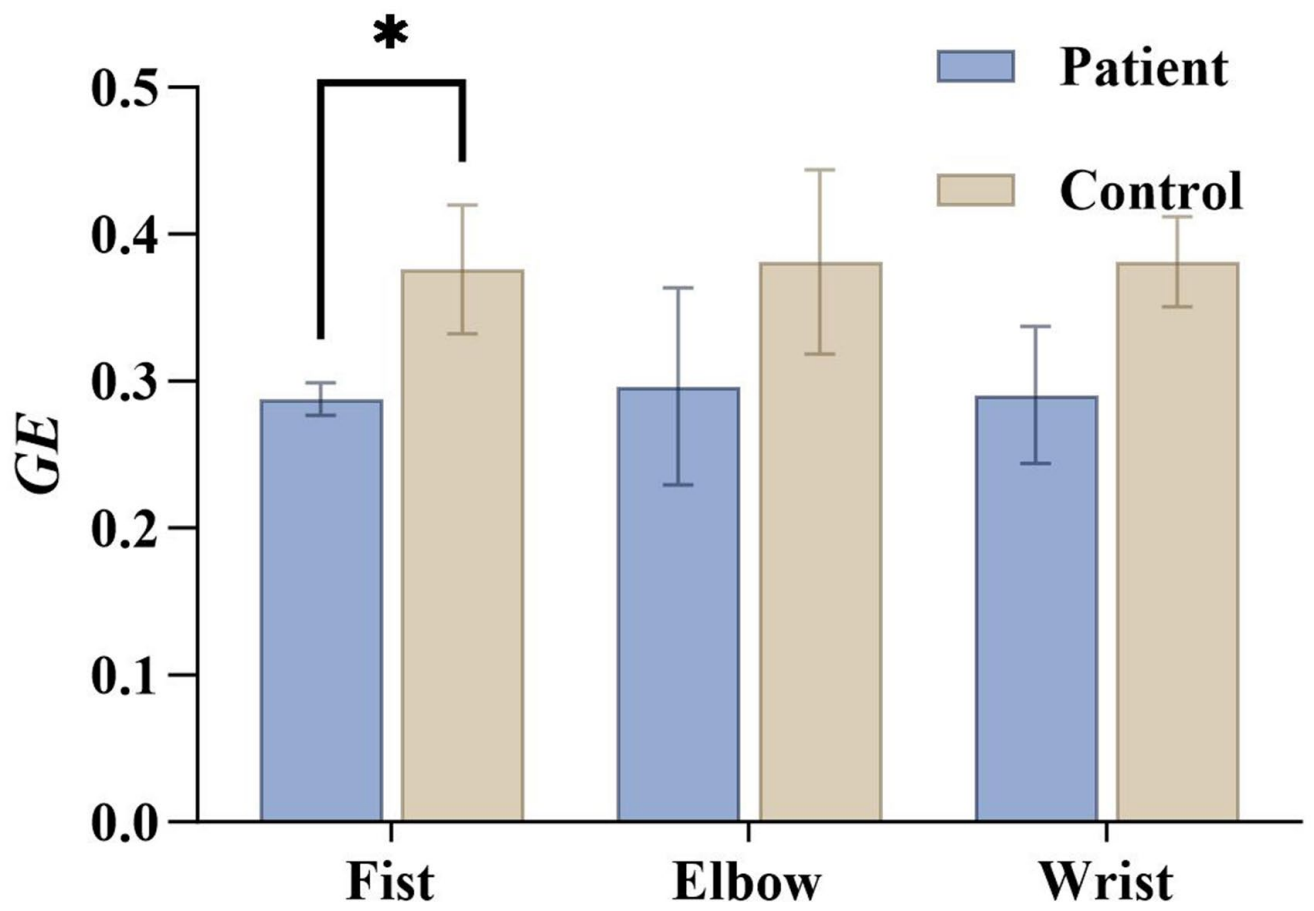
Global efficiency statistics under three actions for the patient group and the healthy group. *GE* indicates global efficiency.

*GE* for the patient group and healthy control group is shown in Figure 10. The patient group exhibited significantly lower global efficiency during the fist-clenching task compared to the healthy control group ( t(14) = 8.865, <0.001 $Cohen^{\prime }s\;d=4.43$ ), indicating reduced overall information transmission efficiency in the patients’ brains and impaired cross-regional coordination. Global efficiency also showed a declining trend during elbow flexion and wrist flexion tasks, though the differences did not reach statistical significance.

**Figure 11:**
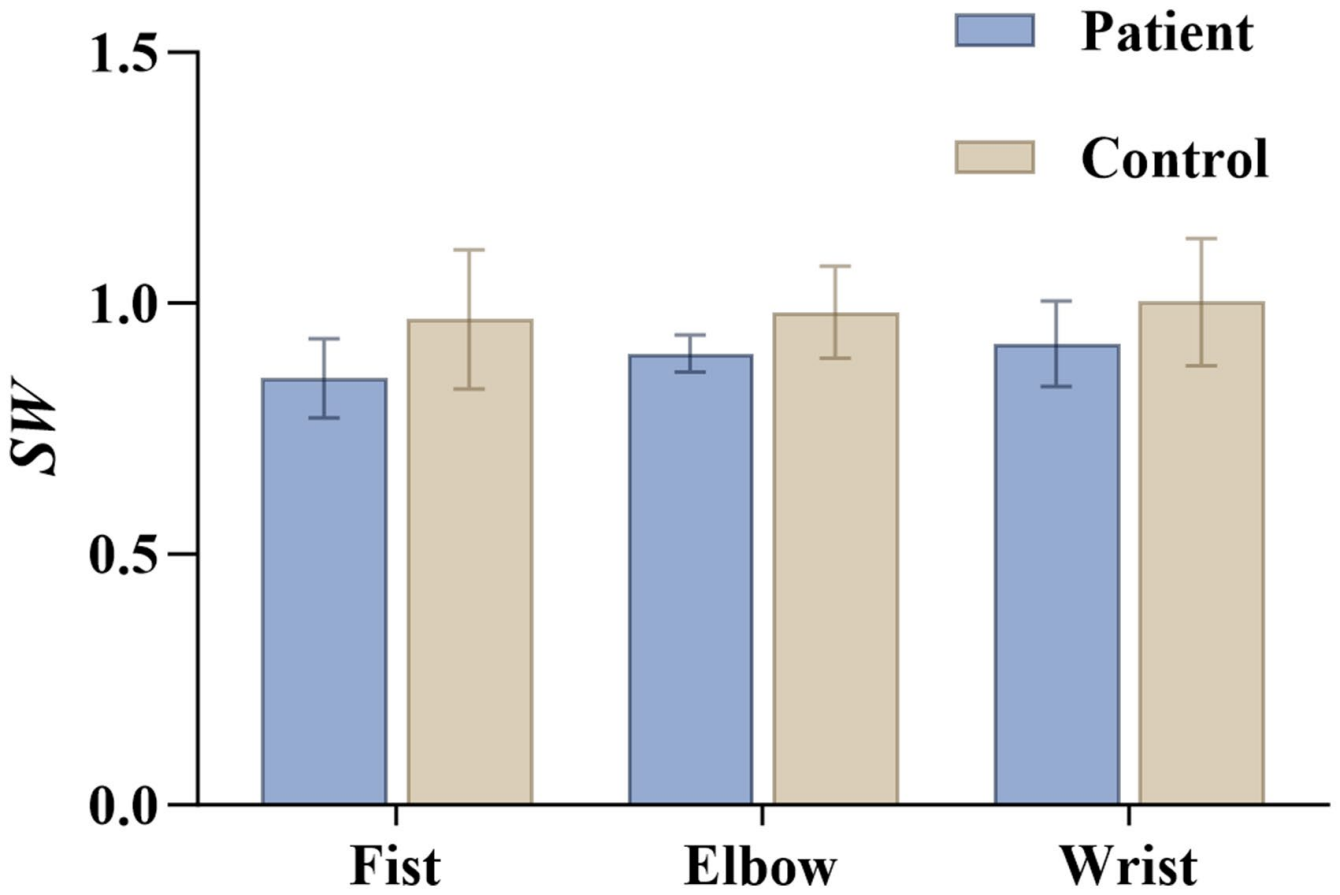
Statistics of small world attributes under three actions in the patient and healthy groups. *SW* indicates small-world property.

*SW* results (Figure 11) indicate no significant differences between the two groups across all three action tasks. This suggests that despite reduced overall and local efficiency in patients, the brain’s overall topological structure retains certain small-world characteristics. This may reflect the inherent robustness of human brain networks.

To further investigate the relationship between network characteristics and spasticity severity, we performed a Spearman rank correlation analysis between the network metrics and the Modified Ashworth Scale (MAS) scores within the patient group. The analysis revealed a significant positive correlation between Global Efficiency (GE) and MAS scores during the fist-clenching task ( ρ = 0.82, p = 0.018). This suggests that patients with higher spasticity scores tended to exhibit higher global efficiency values during this specific motor task. No statistically significant correlations were observed for other metrics or tasks ( p > 0.05).

## 4 Discussion

This study systematically compared the differences in brain functional network characteristics between patients with upper limb spasticity after stroke and healthy individuals during three motor tasks—fist clenching, elbow flexion, and wrist flexion—through EEG collection and analysis. Results revealed that spasticity patients exhibited significant brain network remodeling across different frequency bands and task conditions. Specifically, the patient group exhibited generally lower functional connectivity strength in the alpha and beta bands compared to the healthy group, with notably weakened connections in frontal, parietal, and temporal regions. This aligns with previous findings of impaired alpha and low-beta band networks following stroke (Shim et al., 2023; Snyder et al., 2021; Rosso et al., 2019). Regarding network metrics, the patient group exhibited significant reductions in global efficiency, local efficiency, and clustering coefficient, while small-world properties remained relatively stable (Almeida et al., 2022; De Vico Fallani et al., 2013). These findings reveal that post-stroke spasticity is not merely a manifestation of peripheral neuromuscular dysregulation but more profoundly reflects disturbances and reorganization within the central nervous system’s functional networks.

First, this study found that reduced global efficiency in spasticity patients indicates weakened overall information transmission and integration capabilities within the brain (Figure 10). As a key indicator of cross-regional information transfer capacity, lower global efficiency values suggest patients struggle to effectively integrate neural activity across different brain regions during motor tasks, leading to impaired coordination in movement control. Previous imaging and graph theory studies have demonstrated a close association between reduced global efficiency post-stroke and impaired motor function. This finding has been validated in both resting-state fMRI topological modeling and task-state network analysis (Ward et al., 2003). Our results align with these previous studies, further substantiating global efficiency as an objective indicator reflecting spasticity severity. Furthermore, our correlation analysis revealed a significant positive association between Global Efficiency and MAS scores during the fist-clenching task. This finding implies that within the spasticity cohort, patients with higher clinical spasticity severity may recruit more extensive global network resources to execute the motor task. This phenomenon could reflect a compensatory mechanism where the brain mobilizes a broader range of neural circuits to overcome the increased muscle tone and motor deficits. This significant correlation provides direct evidence that the observed network alterations are not merely generic stroke sequelae but are specifically sensitive to the severity of spasticity.

Secondly, the decline in local efficiency and clustering coefficient reflects impaired robustness and modular organization in information transmission within local networks. Higher local efficiency enables the brain to maintain fault tolerance in information transmission when local networks are damaged, while elevated clustering coefficients indicate strong modular collaboration between brain regions. Our findings reveal significant declines in these metrics among spasticity patients, suggesting disruption in both local information processing and modular structure (Figures 8, 9). This impairment may lead patients to over-rely on compensatory activity in partially preserved brain regions during movement execution, resulting in regionally enhanced functional connectivity and reduced overall efficiency. Similar findings were observed in Rolle C E et al.’s EEG network study, which revealed that post-stroke spasticity patients exhibited lower local efficiency and clustering coefficients than healthy controls, negatively correlated with MAS scores (Rolle et al., 2021). This indicates that central abnormalities in spasticity patients extend beyond isolated regional damage, involving broader disintegration and reorganization of local networks.

The small-world property showed no significant difference between the two groups (Figure 11). This finding indicates that despite impaired network efficiency and modularity in spasticity patients, the overall topological structure of the brain retains a degree of robustness. Small-world networks are considered an efficient topological pattern intermediate between regular and random networks, combining short path lengths with high clustering properties. They form a crucial foundation for the brain’s efficient information processing. Our findings demonstrate that despite extensive functional connectivity impairment following stroke, the brain retains small-world properties to some extent, reflecting compensatory neural remodeling mechanisms. Blaschke et al. observed in an experimental stroke model that small-world properties transiently increased during the subacute phase (potentially reflecting hyperconnectivity or compensatory remodeling), subsequently returning to baseline levels as function recovered. This further supports the brain’s compensatory mechanisms for maintaining or adjusting small-world structures after injury (Sharpee et al., 2016).

Analysis of node degrees further revealed abnormal changes in key hub brain regions among spasticity patients. This study found that the patient group exhibited higher node degrees in electrodes overlying temporal and parietal regions (such as T3 and P3), whereas the healthy group showed higher node degrees in electrodes corresponding to central and frontal regions (Figure 7). This discrepancy suggests that patients may rely on compensatory activation in atypical regions during motor tasks to compensate for deficits in motor cortex function. However, this compensation lacks the coordinated global integration observed in healthy brains, resulting in reduced efficiency. Previous studies have demonstrated that stroke patients exhibit additional activation in non-motor regions such as the prefrontal cortex and cingulate cortex during the early stages of motor tasks (Yu et al., 2024). Thus, our findings not only reveal abnormal compensatory patterns in spasticity patients but also provide new evidence for understanding the central mechanisms underlying spasticity.

From a frequency band perspective, recent neurophysiological models suggest that alpha oscillations facilitate information gating by suppressing irrelevant sensory inputs (Ulanov and Shtyrov, 2022; Ding et al., 2025), while beta oscillations are instrumental in maintaining the current sensorimotor state and modulating top-down inhibitory control (Barone and Rossiter, 2021). The significant reduction in alpha connectivity observed in our spasticity cohort aligns with findings (Shim et al., 2023), supporting the hypothesis that spasticity involves a failure of central inhibitory gating, which may lead to an inability to suppress abnormal stretch reflexes. Similarly, the disrupted beta network suggests impaired cortical modulation of spinal excitability. While some acute-stage studies report hyper-connectivity as a compensatory strategy, our findings in the subacute/chronic phase point toward a ‘network exhaustion’ or maladaptive reorganization. Crucially, however, the cross-sectional design of this study permits only the identification of associations between these network metrics and spasticity. We cannot definitively conclude that these network disruptions are the direct cause of spasticity severity; they may alternatively represent a maladaptive plastic response to long-term sensorimotor restriction. Future longitudinal studies tracking network dynamics are needed to establish causality.

In terms of clinical significance, the findings of this study indicate that brain network metrics can serve as objective tools for quantifying spasticity. Traditional scales suffer from high subjectivity and low resolution, whereas brain network metrics directly reflect the information processing capabilities of spasticity patients at the central nervous system level. Decreases in global efficiency, local efficiency, and clustering coefficient can serve as quantitative parameters for spasticity severity, potentially evolving into novel methods for rehabilitation assessment and treatment monitoring. Furthermore, these metrics provide new targets for rehabilitation interventions. For instance, neuromodulation techniques like transcranial direct current stimulation (tDCS) and repetitive transcranial magnetic stimulation (rTMS) can improve network efficiency by enhancing connectivity between the motor cortex and parietal regions, thereby alleviating spasticity. Brain-computer interface (BCI) training similarly holds promise for reshaping network architecture by promoting synergistic activation across brain regions, ultimately enhancing rehabilitation outcomes.

This study has some limitations that should be acknowledged. First, the sample size was relatively modest, which limits the statistical power and may affect the generalizability of the findings; future studies with larger cohorts are warranted to further validate the results. Second, due to the limited spatial resolution of the 19-channel montage, we focused on sensor-level topological features rather than performing source localization (e.g., LORETA), which requires high-density EEG for reliable anatomical precision. Consequently, deep brain regions such as the basal ganglia and thalamus were not directly assessed; incorporating multimodal approaches (e.g., fMRI) could provide a more comprehensive understanding. Finally, the present work focused on static functional connectivity; future investigations should employ dynamic network analysis (e.g., sliding-window approaches) to capture the temporal variability of brain network reorganization during motor tasks.

## 5 Conclusion

In summary, this study reveals the characteristics of functional network remodeling in patients with upper limb spasticity following stroke across different motor tasks. These changes manifest as decreased global efficiency, local efficiency, and clustering coefficient, alongside significantly weakened functional connectivity in the alpha and beta frequency bands. These findings not only deepen our understanding of the neural mechanisms underlying spasticity but also suggest that brain network metrics hold potential as objective quantitative tools. They provide crucial theoretical foundations and practical references for future personalized rehabilitation interventions and neuromodulation strategies. These discoveries indicate that spasticity development is closely associated with abnormal remodeling of central neural networks and offer potential physiological indicators for its objective assessment. Future research should validate the reliability and sensitivity of brain network metrics as quantitative tools for spasticity using larger samples and multimodal data, while exploring their integration with clinical rehabilitation interventions.

## Data Availability

The raw data supporting the conclusions of this article will be made available by the authors, without undue reservation.

## References

[ref1] Abbas A. K. Azemi G. Ravanshadi S. Omidvarnia A. (2021). An EEG-based methodology for the estimation of functional brain connectivity networks: application to the analysis of newborn EEG seizure. Biomed. Signal Process. Control 63:102229. doi: 10.1016/j.bspc.2020.102229

[ref2] Alaei H. S. Ghoshuni M. Vosough I. (2023). Directed brain network analysis in anxious and non-anxious depression based on EEG source reconstruction and graph theory. Biomed. Signal Process. Control 83:104666. doi: 10.1016/j.bspc.2023.104666

[ref3] Almeida S. R. M. Stefano Filho C. A. Vicentini J. Novi S. L. Mesquita R. C. Castellano G. . (2022). Modeling functional network topology following stroke through graph theory: functional reorganization and motor recovery prediction. Braz. J. Med. Biol. Res. 55:e12036. doi: 10.1590/1414-431X2022e12036, PMID: 35976269 PMC9377533

[ref4] Asadi B. Cuenca-Zaldivar J. N. Nakhostin Ansari N. Ibanez J. Herrero P. Calvo S. (2023). Brain analysis with a complex network approach in stroke patients based on electroencephalography: a systematic review and Meta-analysis. Healthcare 11:666. doi: 10.3390/healthcare11050666, PMID: 36900671 PMC10000667

[ref5] Barone J. Rossiter H. E. (2021). Understanding the role of sensorimotor Beta oscillations. Front. Syst. Neurosci. 15:655886. doi: 10.3389/fnsys.2021.655886, PMID: 34135739 PMC8200463

[ref6] Bistriceanu C. E. Danciu F. A. Cuciureanu D. I. (2023). Cortical connectivity in stroke using signals from resting-state EEG: a review of current literature. Acta Neurol. Belg. 123, 351–357. doi: 10.1007/s13760-022-02102-z, PMID: 36190646

[ref7] De Vico Fallani F. Pichiorri F. Morone G. Molinari M. Babiloni F. Cincotti F. . (2013). Multiscale topological properties of functional brain networks during motor imagery after stroke. NeuroImage 83, 438–449. doi: 10.1016/j.neuroimage.2013.06.039, PMID: 23791916

[ref8] Ding L. Tian X. Ren H. Chen Z. Shu X. Chen S. . (2025). Resting-state EEG associated with clinical measures to predict upper limb motor recovery of subacute stroke. Front. Neurol. 16:1577393. doi: 10.3389/fneur.2025.1577393, PMID: 40917654 PMC12411749

[ref9] Fan Z. Xi X. Wang T. Li H. Maofeng W. Li L. . (2023). Effect of tDCS on corticomuscular coupling and the brain functional network of stroke patients. Med. Biol. Eng. Comput. 61, 3303–3317. doi: 10.1007/s11517-023-02905-z, PMID: 37667074

[ref10] Feigin V. L. Stark B. A. Johnson C. O. (2021). Global, regional, and national burden of stroke and its risk factors, 1990-2019: a systematic analysis for the global burden of disease study 2019. Lancet Neurol. 20, 795–820. doi: 10.1016/S1474-4422(21)00252-0, PMID: 34487721 PMC8443449

[ref11] Imperatori L. S. Betta M. Cecchetti L. Canales-Johnson A. Ricciardi E. Siclari F. . (2019). EEG functional connectivity metrics wPLI and wSMI account for distinct types of brain functional interactions. Sci. Rep. 9:8894. doi: 10.1038/s41598-019-45289-7, PMID: 31222021 PMC6586889

[ref12] Ji G. J. Yu Y. Miao H. H. Wang Z. J. Tang Y. L. Liao W. (2017). Decreased network efficiency in benign epilepsy with Centrotemporal spikes. Radiology 283, 186–194. doi: 10.1148/radiol.2016160422, PMID: 27631414

[ref13] Lance J. W. (1980). The control of muscle tone, reflexes, and movement: Robert Wartenberg lecture. Neurology 30, 1303–1313. doi: 10.1212/WNL.30.12.1303, PMID: 7192811

[ref14] Li J. Zhang Q. Wang J. Xiong Y. Zhu W. (2024). Network efficiency of functional brain connectomes altered in type 2 diabetes patients with and without mild cognitive impairment. Diabetol. Metab. Syndr. 16:247. doi: 10.1186/s13098-024-01484-9, PMID: 39402665 PMC11476597

[ref15] Lin Y. Jiang Z. Zhan G. Su H. Kang X. Jia J. (2023). Brain network characteristics between subacute and chronic stroke survivors in active, imagery, passive movement task: a pilot study. Front. Neurol. 14:1143955. doi: 10.3389/fneur.2023.1143955, PMID: 37538258 PMC10395333

[ref16] Ma X. Jiang G. Fu S. Fang J. Wu Y. Liu M. . (2018). Enhanced network efficiency of functional brain networks in primary insomnia patients. Front. Psych. 9:46. doi: 10.3389/fpsyt.2018.00046, PMID: 29515469 PMC5826384

[ref17] Milani G. Antonioni A. Baroni A. Malerba P. Straudi S. (2022). Relation between EEG measures and upper limb motor recovery in stroke patients: a scoping review. Brain Topogr. 35, 651–666. doi: 10.1007/s10548-022-00915-y, PMID: 36136166 PMC9684227

[ref18] Pandyan A. D. Johnson G. R. Price C. I. M. Curless R. H. Barnes M. P. (1999). A review of the properties and limitations of the Ashworth and modified Ashworth scales as measures of spasticity. Clin. Rehabil. 13:5404. doi: 10.1191/026921599677595404, PMID: 10498344

[ref19] Pion-Tonachini L. Kreutz-Delgado K. Makeig S. (2019). ICLabel: an automated electroencephalographic independent component classifier, dataset, and website. NeuroImage 198, 181–197. doi: 10.1016/j.neuroimage.2019.05.026, PMID: 31103785 PMC6592775

[ref20] Rolle C. E. Baumer F. M. Jordan J. T. Berry K. Garcia M. Monusko K. . (2021). Mapping causal circuit dynamics in stroke using simultaneous electroencephalography and transcranial magnetic stimulation. BMC Neurol. 21:280. doi: 10.1186/s12883-021-02319-0, PMID: 34271872 PMC8283835

[ref21] Rosso O. A. Montani F. Baravalle R. Guisande N. Granado M. (2019). Characterization of visuomotor/imaginary movements in EEG: an information theory and complex network approach. Front. Phys. 7:115. doi: 10.3389/fphy.2019.00115

[ref22] Rubinov M. Sporns O. (2011). Weight-conserving characterization of complex functional brain networks. Neuroimage 56, 2068–2079. doi: 10.1016/j.neuroimage.2011.03.069, PMID: 21459148

[ref23] Shafiei S. B. Shadpour S. Shafqat A. (2024). Mental workload evaluation using weighted phase lag index and coherence features extracted from EEG data. Brain Res. Bull. 214:110992. doi: 10.1016/j.brainresbull.2024.110992, PMID: 38825253 PMC11734752

[ref24] Sharpee T. O. Destexhe A. Kawato M. Sekulić V. Skinner F. K. Wójcik D. K. . (2016). 25th annual computational neuroscience meeting: CNS-2016. BMC Neurosci. 17:54. doi: 10.1186/s12868-016-0283-6, PMID: 27534393 PMC5001212

[ref25] Shim M. Choi G. Y. Paik N. J. Lim C. Hwang H. J. Kim W. S. (2023). Altered functional networks of alpha and low-Beta bands during upper limb movement and association with motor impairment in chronic stroke. Brain Connect. 13, 487–497. doi: 10.1089/brain.2021.0070, PMID: 34269616

[ref26] Snyder D. B. Schmit B. D. Hyngstrom A. S. Beardsley S. A. (2021). Electroencephalography resting-state networks in people with stroke. Brain Behav. 11:e02097. doi: 10.1002/brb3.2097, PMID: 33759382 PMC8119848

[ref27] Su H. Zhan G. Lin Y. Wang L. Jia J. Zhang L. . (2025). Analysis of brain network differences in the active, motor imagery, and passive stoke rehabilitation paradigms based on the task-state EEG. Brain Res. 1846:149261. doi: 10.1016/j.brainres.2024.149261, PMID: 39396567

[ref28] Ulanov M. Shtyrov Y. (2022). Oscillatory beta/alpha band modulations: a potential biomarker of functional language and motor recovery in chronic stroke? Front. Hum. Neurosci. 16:940845. doi: 10.3389/fnhum.2022.940845, PMID: 36226263 PMC9549964

[ref29] Van Kaam R. C. Van Putten M. J. a. M. Vermeer S. E. Hofmeijer J. (2018). Contralesional brain activity in acute ischemic stroke. Cerebrovasc. Dis. 45, 85–92. doi: 10.1159/000486535, PMID: 29510399

[ref30] Wang T. Wang C. Chen K. Yang D. Xi X. Kong W. (2024). Evaluating stroke rehabilitation using brain functional network and corticomuscular coupling. Int. J. Neurosci. 134, 234–242. doi: 10.1080/00207454.2022.2099386, PMID: 35815432

[ref31] Wang J. Wang X. Xia M. Liao X. Evans A. He Y. (2015). GRETNA: a graph theoretical network analysis toolbox for imaging connectomics. Front. Hum. Neurosci. 9:386. doi: 10.3389/fnhum.2015.00386, PMID: 26175682 PMC4485071

[ref32] Ward A. B. (2008). Spasticity treatment with botulinum toxins. J. Neural Transm. 115, 607–616. doi: 10.1007/s00702-007-0833-2, PMID: 18389166

[ref33] Ward N. S. Brown M. M. Thompson A. J. Frackowiak R. S. (2003). Neural correlates of motor recovery after stroke: a longitudinal fMRI study. Brain 126, 2476–2496. doi: 10.1093/brain/awg245, PMID: 12937084 PMC3717457

[ref34] Watts D. J. Strogatz S. H. (1998). Collective dynamics of ‘small-world’networks. Nature 393, 440–442. doi: 10.1038/30918, PMID: 9623998

[ref35] Wissel J. Manack A. Brainin M. (2013). Toward an epidemiology of poststroke spasticity. Neurology 80, S13–S19. doi: 10.1212/WNL.0b013e3182762448, PMID: 23319481

[ref36] Yan Y. Zhao A. Ying W. Qiu Y. Ding Y. Wang Y. . (2021). Functional connectivity alterations based on the weighted phase lag index: an exploratory electroencephalography study on Alzheimer's disease. Curr. Alzheimer Res. 18, 513–522. doi: 10.2174/1567205018666211001110824, PMID: 34598666

[ref37] Yu P. Dong R. Wang X. Tang Y. Liu Y. Wang C. . (2024). Neuroimaging of motor recovery after ischemic stroke - functional reorganization of motor network. Neuroimage Clin. 43:103636. doi: 10.1016/j.nicl.2024.103636, PMID: 38950504 PMC11267109

[ref38] Zhang H. Zhou Q. Q. Chen H. Hu X. Q. Li W. G. Bai Y. . (2023). The applied principles of EEG analysis methods in neuroscience and clinical neurology. Mil. Med. Res. 10:67. doi: 10.1186/s40779-023-00502-7, PMID: 38115158 PMC10729551

